# Awareness of the Effect of Vitamin B12 Deficiency on the Nervous System Among the General Population in Taif, Saudi Arabia

**DOI:** 10.7759/cureus.49343

**Published:** 2023-11-24

**Authors:** Raneem M Alqurashi, Aliah Aladwani, Teef H Alosaimi, Deema B Yousef, Maha H Alkhaldi, Mona G Amer

**Affiliations:** 1 Medicine, Taif University, Taif, SAU; 2 Anatomy, Taif University, Taif, SAU

**Keywords:** taif, saudi arabia, deficiency, awareness, vitamin b12

## Abstract

Background: Vitamin B12 is important for the health of the nervous system, its deficiency leads to various neurological manifestations such as visual problems, ataxia, peripheral neuropathy, dementia, etc. The deficiency can be caused by malnutrition, malabsorption, or increased demand. Early detection is important for the control and prevention of complications.

Method: In December 2021, a population-based cross-sectional survey was carried out among Saudi males and women at least 18 years old. There were 383 participants in the sample. An electronic survey distributed over social media was used to collect the data. SPSS version 28 was used to analyze the data.

Results: The majority of the respondents were female (88%). Most participants were aged 18-25 years (44%). Regarding participants' awareness and knowledge of vitamin deficiency, 64% were aware of vitamin B12 deficiency. 41.7% of participants knew about food sources of vitamin B12, 29.0% knew how to prevent it, and 30.0% took vitamin B12 supplements. (92.2%) of the participants were not following vegetarian or vegan diets. Difficulty concentrating accounted for the highest number of reported symptoms by the respondents (53.8%).

Conclusion: This study recommends a scientific approach encouraging patients to self-report their B12 deficiency in medical institutions. In addition, a study about the relationship between B12 deficiency and other neurodegenerative disorders is also a recommendation of this study.

## Introduction

Vitamin B12 (cobalamin) is a water-soluble vitamin bound to proteins in food intake. With the help of intrinsic factor (IF) binding to it (a protein produced by the stomach's parietal cells), the complex is absorbed in the distal ileum and enters the bloodstream. After completing its purpose, it's excreted in the feces [1]. Vitamin B12 is required for the development, myelination, and function of the central nervous system, healthy red blood cell formation, and DNA synthesis [2].

Vitamin B12 deficiency is one of the most common vitamin deficiencies across many countries, causing major public health concerns since it can lead to many serious health problems if not diagnosed and treated properly and early enough [3]. Food sources rich in vitamin B12 include all animal-source foods, i.e., meat, dairy, fish, and eggs. Also, fortified foods, such as breakfast cereals. All dairy products, i.e., milk, yogurt, and cheese [4].

The recommended dietary intake (RDA) of Vitamin B12 daily changes depending on the person's age. For instance, healthy people aged 1-3 years may require 0.9 mcg daily to not be deficient and manifest symptoms, whereas those 14 years and older may need 2.4 mcg daily. Other factors that affect the vitamin's amount or adjust the body's need to become above the normal population average should be considered during evaluation, such as pregnancy needs 2.6 mcg daily and breastfeeding women need 2.8 mcg daily since their requirement heightened [5].

The most common effect of vitamin B12 deficiency is megaloblastic anemia, which usually presents as fatigue, pale skin, weight loss, and palpitation. It is also combined with neurological manifestations such as visual problems, paresthesia, ataxia, spasticity, peripheral neuropathy, dementia, depression, paranoia, and autonomic dysfunction [4].

Lack of vitamin B12 can be caused by malnutrition, malabsorption, or increased demand. Conditions associated with vitamin B12 deficiency include pernicious anemia, an irreversible autoimmune disease that affects the gastric mucosa and results in gastric atrophy and malabsorption of dietary vitamin B12 [6]. Atrophic gastritis is an autoimmune condition affecting 2% of the general population; it decreases the production of intrinsic factors and secretion of hydrochloric acid in the stomach and thus decreases the absorption of vitamin B12 [7]. Also, Helicobacter pylori infection, especially in older adults, has the same effects as Atrophic gastritis, possibly because this bacterium causes inflammation that leads to malabsorption of vitamin B12 from food [8]. Also, surgical procedures in the gastrointestinal tract, such as gastrectomy, can cause a complete or partial loss of cells that secrete hydrochloric acid and cells that secrete intrinsic factors. Vegetarians who occasionally eat animal products and vegans who never eat any animal products (e.g., dairy products, eggs) have a higher risk of developing vitamin B12 deficiency [9]. 

Diagnoses can be established by taking a detailed history, physical examination, and hematological tests. It is reported that 20-30% of cases of vitamin B12 deficiency had neurological manifestations with no signs of anemia [1]. Folic Acid and vitamin B12 are extremely important to reduce plasma homocysteine levels [10].

Traditionally, clinical vitamin B12 deficiency has been treated with intramuscular injections of crystalline vitamin B12 at 1 mg weekly for eight weeks, followed by 1 mg monthly for life [11]. Because crystalline formulations of vitamin B12 are better absorbed than naturally occurring vitamin B12, patients over 50 and strict vegetarians should consume vitamin B12-fortified foods and supplements together [11].

This research aims to assess the awareness of vitamin B12 deficiency among the Saudi Arabian population of the Taif region. This knowledge would benefit the research field in developing more targeted public health awareness programs to prevent, early detect, and treat deficient people, which will hugely reduce the side effects and lead to good outcomes.

## Materials and methods

This study is a cross-sectional study and was conducted from October 2021 to February 2022. This research was sent for approval by a local ethical committee. The committee is accredited by the National Committee for Bioethics with IRB No. (HAO-02-T-105). The estimated sample size was 400 participants with a 5% margin of error using the Raosoft program. 

The inclusion criteria were all Saudi population in the Taif region, males and females from 18 to 65 years old, in normal health conditions and with chronic diseases. Exclusion criteria were pregnant women and people under 18 years old. 

Data was collected through an electronic questionnaire distributed among the Saudi population in the Taif region through social media (WhatsApp, Twitter, and Telegram). The questionnaire included data about personal characteristics (i.e., age, gender, educational level, and employment status). It included participants' awareness and knowledge about vitamin B12 (i.e., importance, effects, food sources, symptoms, signs, and complications due to vitamin B12 deficiency, and the need for awareness about vitamin B12 deficiency).

The data were coded, tabulated, and analyzed using SPSS version 28. Qualitative data were expressed as numbers and percentages, and the Chi-squared test (χ2) was applied to test the relationship between variables.

## Results

The majority of the respondents, n=383, were female (88%) of total respondents. The respondents were categorized into three major age groups, starting with 18-25-year-olds, who comprised the majority (44%) of the sample. Most respondents were students (39%) and workers comprised 30% (Table 1).

**Table 1:** Demographic features of the participants The data has been represented as n Count of males and females (383). (%) = Relative Frequency

Demographic features		Count (n=383)	Relative Frequency (%)
Gender	Male	45	11.7
	Female	338	88.3
Age	18-25	167	43.6
	26-35	50	13.1
	36-45	91	23.8
	above 46	75	19.6
Nationality	Saudi	355	92.7
	Non-Saudi	28	7.3
Occupation	Student	149	38.9
	Worker	118	30.8
	Other	116	30.3

In Table 2, 88% of the respondents were aware of vitamin B12. Among the studied population, (64.5%) knew the importance of vitamin B12 intake. Half of the respondents knew the effects of B12 deficiency on the nervous system. (41.7%) were acquainted with the food sources for vitamin B12, (29.0%) of participants knew how to prevent it, and (30.0%) were on vitamin B12 supplements. While (92.2%) do not follow vegetarian or vegan diets.

**Table 2:** Percentage of participants’ awareness and knowledge about vitamin B12 deficiency.

Item:	Answer	Result. *n* (%)
Have you ever heard about Vitamin B12?	Yes	336 (87.7%)
	No	47 (12.3%)
Do you know the effects of vitamin B12 deficiency?	Yes	246 (64.1%)
	No	138 (35.9%)
Do you know the effects of vitamin B12 deficiency on the nervous system?	Yes	206 (53.8%)
	No	177 (46.2%)
Do you know the importance of vitamin B12?	Yes	247 (64.5%)
	No	136 (35.5%)
Are you a strict vegetarian or vegan?	Yes	30 (7.8%)
	No	353 (92.2%)
Do you know any food source for vitamin B12?	Yes	160 (41.7%)
	No	223 (58.3%)
Do you know how to prevent vitamin B12 deficiency?	Yes	111 (29.0%)
	No	272 (71.0%)
Do you take any supplements for vitamin B12?	Yes	115 (30.0%)
	No	268 (70.0%)

**Figure 1:**
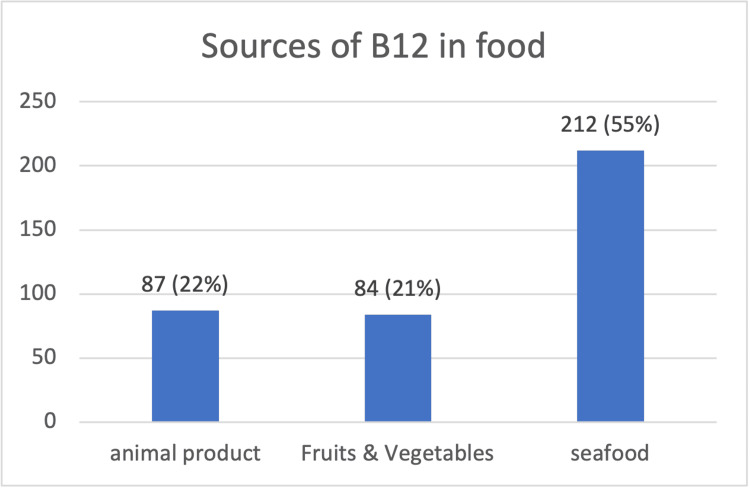
Distribution of B12 food sources chosen by respondents n (%)

Seafood was the largest source of vitamin B12 mentioned by the respondents, accounting for 55% of all the sources; fruits and vegetables were the least sources of B12 at 22% (Figure 1).

Action toward vitamin B12 levels

**Figure 2:**
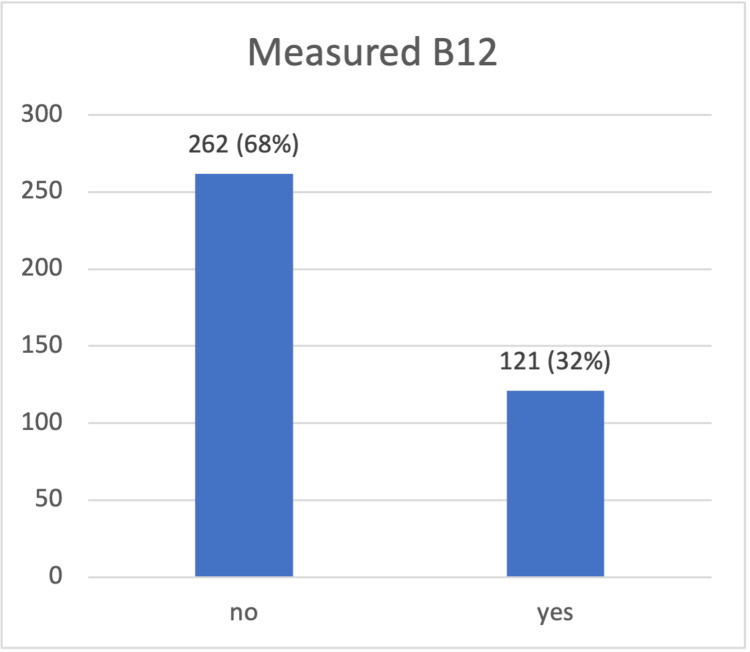
Measure B12 n (%)

The results show that most (68%) respondents had not taken the necessary steps to measure their B12 levels (Figure 2).

**Figure 3:**
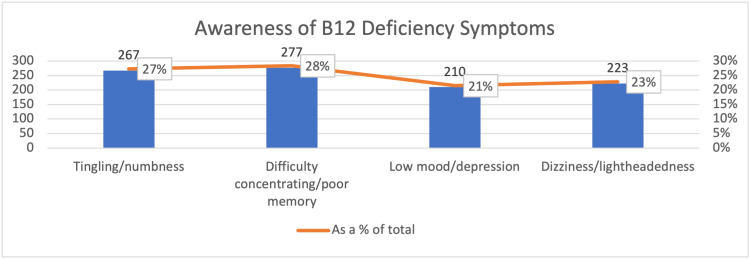
Awareness of B12 Deficiency Symptoms % = Respondents

Difficulty concentrating/poor memory had the highest level of awareness at 28%, while low mood/depression accounted for 21% of symptoms’ awareness (Figure 3).

Relationship of sociodemographic characteristics

It appears from Table 3 that none of the demographic features has shown any statistically significant association with the knowledge of the effects of vitamin B12 on the nervous system.

**Table 3:** The sociodemographic characteristics among people who know the effects of vitamin B12 on the nervous system. P -value < 0.05

Demographic features		Knowledge			P -value
		Yes *n* (%)	No *n* (%)		
Gender	Male	21(5.4%)		24(6.2%)	0.308
	Female	185(48.3%)		153(39.9%)	
Age	18-25	85 (22.1%)		82 (21.4%)	0.170
	26-35	26 (6.7%)		24 (6.2%)	
	36-45	46 (12.0%)		45 (11.7%)	
	above 46	49 (12.7%)		26 (6.7%)	
Nationality	Saudi	194 (50.6%)		161 (42.0%)	0.228
	Non Saudi	12 (3.1%)		16 (4.1%)	
Occupation	Student	79 (20.6%)		70 (18.2%)	0.417
	Worker	69 (18.0%)		49 (12.7%)	
	Other	58 (15.1%)		58 (15.1%)	

## Discussion

This study aimed to establish awareness of the effects of vitamin B12 deficiency on the nervous system among the general population in Taif, Saudi Arabia. Factors involving awareness and knowledge were considered during the data collection and analysis. Gender and other demographic factors were generalized as this study focused on the correlational analysis of the three independent variables: awareness of B12 deficiency, measurement of B12 levels, and prevalence of symptoms. 

This study was conducted by the Medical Department at Taif University in Saudi Arabia, and this explains the high percentage of respondents being students. Age and gender were the main demographic factors that this study considered, as illustrated in the results. The majority of 383 volunteers who participated were female (88%), and the age group that contained the most respondents was 18-25 years (44%) (Table 1).

In this study, the level of awareness of the effects of vitamin B12 deficiency among respondents was relatively high (64%) (Table 2). While another Saudi study was conducted to evaluate the level of awareness of vitamin B12 among Saudi Arabian residents, 78% of the respondents knew about the importance of vitamin B12 and the effects of its deficiency [12].

It was assessed whether participants were aware of B12 deficiency symptoms such as tingling/numbness, difficulty concentrating or poor memory, low mood or depression, and dizziness or lightheadedness. According to the respondents' reports, (28%) difficulty concentrating was the most prevalent symptom (figure 3). These symptoms are consistent with the description provided by Pawlak et al. of various B12-related symptoms, including mental illness, tremors, sleep loss, difficulty concentrating, and other nervous system complications, making B12 deficiency a commonly misdiagnosed condition. In contrast to Shah et al.'s research of blood donors in a rural tertiary hospital in India, it was discovered that about 35.4% of the participants knew some of the symptoms of B12 deficiency [13]. 

In the present study, 41.7% of participants were aware of rich food sources for vitamin B12. (55.3%). The respondents chose seafood as the primary source of food for vitamin B12 (figure 1). Compared to a previous study, only (16.2%) knew vitamin B12-rich food sources [14].

Alshammari et al. also conducted a study on vitamin B12 deficiency and the knowledge and practice of physicians with regard to screening for B12 deficiency among diabetic patients in Riyadh, Saudi Arabia [15]. The research by Alshammari et al. was particularly interesting because the prevalence of diabetes and the commencement of treatment using metformin was reported to increase the risk of B12 deficiency.

Limitations

The success of this study was flawed by several factors, including difficulty in accessing non-student respondents. In addition, this study sought to acquire respondents' sensitive personal data. Lack of cooperation was also observed with some respondents, especially the male, leading to a high number of female respondents proportionally, some of whom eventually agreed to cooperate. Research on vitamin B12 deficiency is very limited in Saudi Arabia and internationally, making it hard to review and analyze literature effectively.

Recommendations

This study, therefore, recommends a scientific approach to encourage patients in medical institutions to self-report their vitamin B12 levels. In addition, a study about the relationship between vitamin B12 deficiency and other neurodegenerative disorders is also a recommendation for this study.

## Conclusions

This study was conducted in Taif, Saudi Arabia. The sample size for this study was 383 respondents who were mainly settled in Taif city. The study evaluated awareness and knowledge of the symptoms of Vitamin B12 deficiency on the nervous system, including tingling/numbness, difficulty concentrating/poor memory, low mood/depression, and dizziness/lightheadedness. The level of awareness and knowledge of the effect of vitamin B12 deficiency on the nervous system shows no association with demographic features in this study. In conclusion, there is a relatively high level of awareness about B12 deficiency among the respondents in the study population.
